# Nanomaterials in Targeting Cancer Stem Cells for Cancer Therapy

**DOI:** 10.3389/fphar.2017.00001

**Published:** 2017-01-18

**Authors:** Weiwei Qin, Guan Huang, Zuanguang Chen, Yuanqing Zhang

**Affiliations:** Institute of Medical Instrument and Application, School of Pharmaceutical Sciences, Sun Yat-Sen UniversityGuangzhou, China

**Keywords:** cancer stem cells, multidrug resistance, unlimited proliferation, nanomaterials, targeted therapies

## Abstract

Cancer stem cells (CSCs) have been identified in almost all cancers and give rise to metastases and can also act as a reservoir of cancer cells that may cause a relapse after surgery, radiation, or chemotherapy. Thus they are obvious targets in therapeutic approaches and also a great challenge in cancer treatment. The threat presented by CSCs lies in their unlimited proliferative ability and multidrug resistance. These findings have necessitated an effective novel strategy to target CSCs for cancer treatment. Nanomaterials are on the route to providing novel methods in cancer therapies. Although, there have been a large number of excellent work in the field of targeted cancer therapy, it remains an open question how nanomaterials can meet future demands for targeting and eradicating of CSCs. In this review, we summarized recent and highlighted future prospects for targeting CSCs for cancer therapies by using a variety of nanomaterials.

## Introduction

With the financial support of government and society for cancer research, progress has been made in the development of innovative strategies for cancer therapy. However, Cancer still remains one of the deadliest diseases affecting our health, cancer relapse and metastasis are common in patients accepting traditional chemotherapy or radiotherapy. The failure of traditional therapies may be ascribed to a relatively rare subpopulation of cancer cells exist in tumor, called cancer stem cells (CSCs). Since Bonnet and Dick (1997) isolated a small portion of leukemia-initiating cells with features similar to stem cells, researches seemed to focus on isolating CSCs by specific identifying markers. Further researches of other tumor types have identified CSCs in almost all cancers, including prostate (Collins et al., 2005; Maitland and Collins, 2008; Lang et al., 2009), lung (Eramo et al., 2007), colon (O'Brien et al., 2007; Ricci-Vitiani et al., 2007), pancreatic (Hermann et al., 2007; Li et al., 2007), gastric (Fukuda et al., 2009), breast (Al-Hajj et al., 2003), glioma (Galli et al., 2004; Bao et al., 2006), and brain (Hemmati et al., 2003; Singh et al., 2003) cancers. These CSCs exhibit several characteristics, including self-renewal, differentiation into multiple cell types, expression of ATP-binding cassette (ABC) pumps that enable them to resist chemotherapeutic agents, and ionizing radiations.

A variety of nanomaterial, such as DNA (e.g., origami and tetrahedron), carbon (e.g., graphene and nanodiamond), noble metal (e.g., gold and silver nanoparticles), organic polymers, and liposome nanoparticles, with various sizes and modifications to their surfaces can be easily prepared and offer promising means for developing solutions in CSC therapy (Tomuleasa et al., 2012; Orza et al., 2013). Nanomaterial is on the route to providing novel breakthroughs in targeted therapy. During the past decade, nanotechnology and nanomaterial have been widely integrated in biomedical research, providing new strategies for cell imaging (Huang et al., 2006; Kong et al., 2012; Li et al., 2014), siRNA and drug delivery (Panyam and Labhasetwar, 2003; Lee et al., 2010; Malmsten, 2013), and targeted cancer therapy (Brannon-Peppas and Blanchette, 2004; Loo et al., 2005; Bild et al., 2006). All these mentioned should be attributed to the unique properties of these nanomaterial, such as high surface to volume ratio, easiness to be modified, unique optical properties, quantum-size effects (Whitesides et al., 1991). Taking advantage of and combine the excellent properties of various nanomaterials will further provide better solutions for targeted and controlled elimination of CSCs in the future. The purpose of this review is to summarize recent progress in the applications of various nanomaterials for targeting CSCs.

## The biology of cancer stem cells

**Figure 1:**
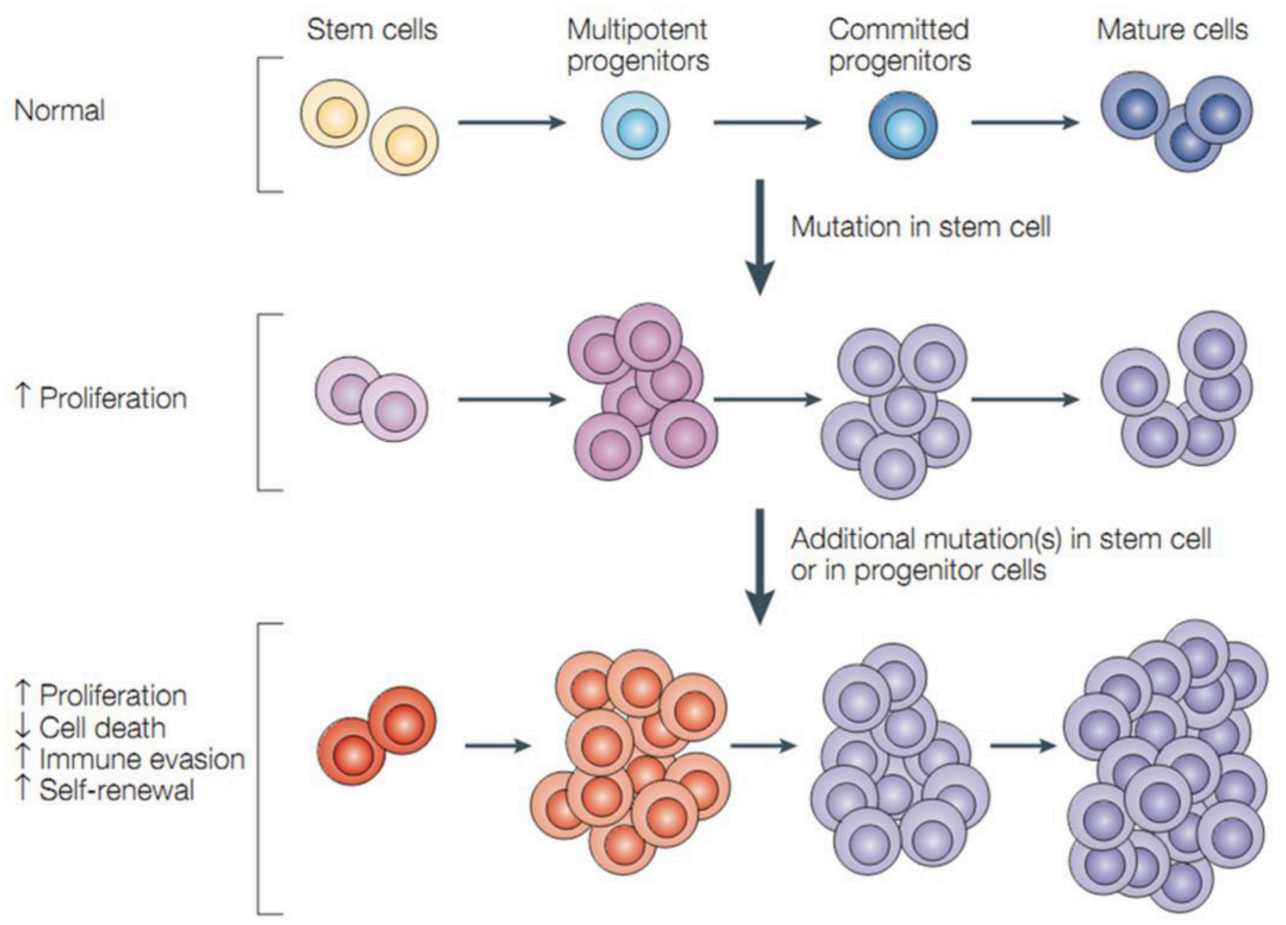
**Cancer stem cells and tumor progression**. Normal stem cells give rise to multipotent progenitor cells, committed progenitors and mature, differentiated cells. Mutations in a stem cell give rise to a stem cell with aberrant proliferation and result in a pre-malignant lesion. Additional mutations lead to the acquisition of further increased proliferation, decreased apoptosis, evasion of the immune system, and further expansion of the stem-cell compartment that is typical of malignant tumors (Dean et al., 2005).

Cells that have the ability to self-renewal and generate mature cells of a specific tissue through differentiation are defined as stem cells. However, tumors may often result from the conversion of normal stem cells, and similar self-renewal can be regulated between stem cells and cancer cells including CSCs—a rare, phenotypically distinct subset of cells that have the capacity to form new tumors (Hamburger and Salmon, 1977; Figure 1). Recent studies indicated that normal stem cells in hematopoietic system are the targets of transforming mutations, and cancer cell proliferation is driven by CSCs. CSC and normal stem cell share a lot of properties. And the most important one is that both of them have unlimited potential (Reya et al., 2001) for self-renewal that promote tumorigenesis and give rise to new (normal or abnormal) tissues. Moreover, CSCs (self-renewal either inherent or acquired) can produce cells that lack long-term self-renewal ability but preserve dividing capability. Thus, CSCs can be thought of as tumorigenic cells that go through an anomalous and scanty regulated process of tumorigenesis, which is similar to what normal stem cells do.

## The necessity of targeting cancer stem cells

Cancer cells can acquire resistance to conventional approaches for cancer treatment such as chemotherapy and radiotherapy (Dean et al., 2005; Eyler and Rich, 2008) by a variety of mechanisms (Dean et al., 2005), including the mutation or overexpression of the drug target, inactivation of the drug, or elimination of the drug from the cell. Drug resistance and cancer metastasis are the two primary problems for the therapy of cancer. Recent studies indicated that endothelial cells can protect normal stem cells and cancer cells from radiation damage (Garcia-Barros et al., 2003; Bao et al., 2006; Diehn and Clarke, 2006). On the basis of CSCs concept, an alternative model posits that the CSCs are naturally resistant to chemotherapy through their quiescence, ABC-transporter expression, and their capacity for DNA repair as they self-renewal, which allow them to expand the population of tumor cells following with chemotherapy or radiotherapy. Furthermore, the survival of residual CSCs is thought to be one of the factors that drives the onset of tumor recurrence, distant metastasis, and drug-resistance, which is a significant clinical problem for the effective treatment of cancer. Conventional chemotherapeutic agents are not only uneasily to accept but also unable to destroy the drug-resistant CSCs, thus it demands a novel approach for cancer therapy. If the chemotherapeutic agents used can efficiently target against CSCs (Stupp and Hegi, 2007), then it might be more effective in killing them. Since more and more efficiently new diagnostic markers (such as, CD44+, CD90+, CD133+, and so on) and therapeutic targets expressed by the stem cells have been found, solid CSCs can be identified prospectively and isolated efficiently. Thus, CSC-based therapies may don't remit cancers at the beginning, but they may eventually cure cancers successfully.

## Applications of nanomaterials for CSC targeting

**Scheme 1:**
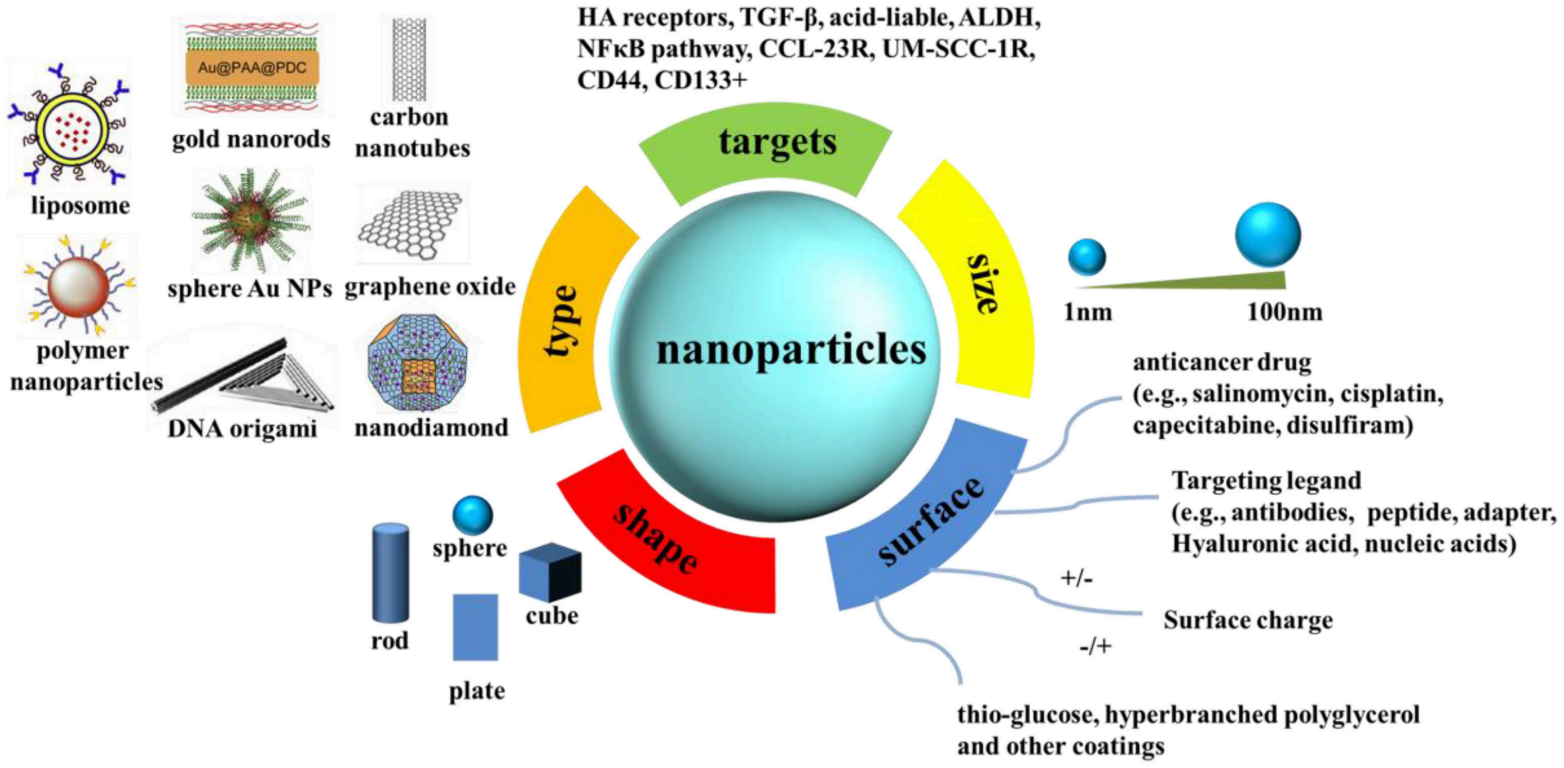
**The mechanisms of the engineered nanoparticles for drug delivery in cancer stem cell therapy**. A summary of nanoparticles that have been explored as carriers for drug delivery in cancer stem cell therapy, together with illustrations of biophysicochemical properties.

Nanomaterials have attracted much attention during the past few decades and will attract more attention in the future owing to their unique optical, chemical, and electronic properties (Manchikanti and Bandopadhyay, 2010; Chen et al., 2013). On the basis of these unique properties, they have been applied in a wide spectrum of fields, including catalysis (Thompson, 2007; Luo et al., 2010; Diao and Cao, 2011; Zheng et al., 2011; Li et al., 2015), plasmonic imaging (Li et al., 2013; Peng et al., 2015), biochemical sensors (Orza et al., 2010; Zheng et al., 2011; Qin et al., 2015; Xu Y. et al., 2015), tumor cell detection (Lu et al., 2010), targeted therapy (Kumar et al., 2012), and so on. The unique characteristics of nanomaterials mainly benefit from their high surface to volume ratio compared with their respective bulk counterparts. The large quantities of surface atoms of nanomaterials enable them to have outstanding surface properties that can be utilized for the modification of anti-cancer drugs, various active agents, and targeting molecules commonly used in cancer therapy. Scheme 1 summarizes the mechanisms of the engineered nanoparticles for drug delivery in cancer stem cell therapy. In combination with the latest findings in the area of CSC researches, nanomaterials will bring new opportunities in detecting and targeting of CSCs.

## Carbon nanomaterials in CSC targeting

During the last decade, carbon nanotechnology has achieved rapid development, allotropes of carbon, especially graphene, diamond and carbon nanotubes, have found a wide range of applications in industry and biomedicine. Carbon nanomaterials have also attracted extensive attention from clinical scientists in frontier research and they were used as potential agents in anticancer therapies.

## Graphene oxide

Graphene began to attract widespread attention since single layers of graphite were obtained by Novoselov et al. (2004) and it was regarded as the most promising material for transistor production that could replace traditional semiconducting materials. Graphene oxide (GO) is a graphene derivative with carbon atoms linked to oxygen functional groups which confers an extraordinary chemical versatility. Thus, the surface of graphene can be easily modified with various biochemical molecules and agents of interest, which enable graphene an excellent carrier of drugs or nucleic acids for targeted cancer therapies.

**Figure 2:**
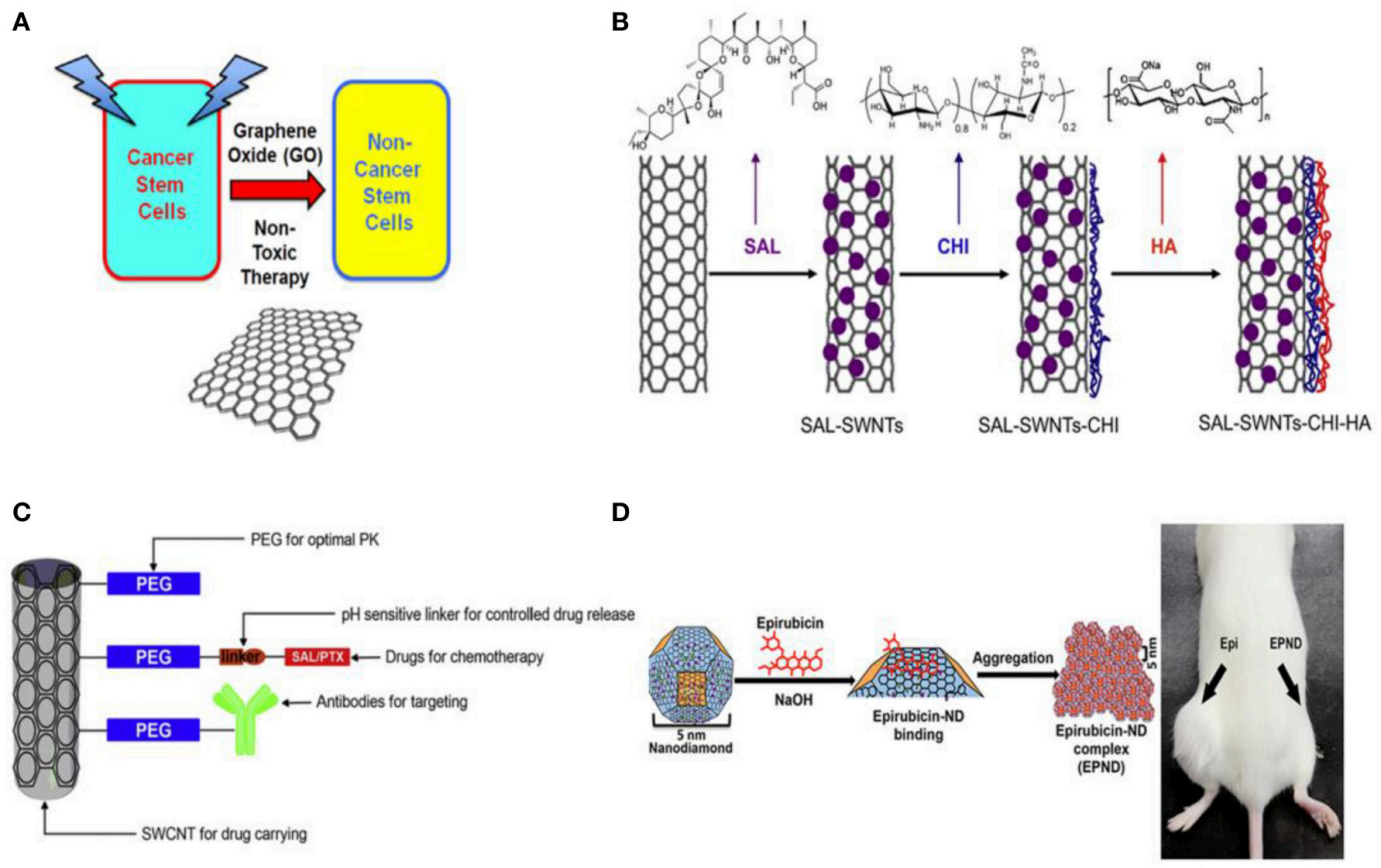
**(A)** Graphene oxide (GO): Targeting cancer stem cells with differentiation-based nano-therapy. The current mechanistic studies suggest that GO could directly be used as a therapeutic for targeting cancer stem cells (CSCs), because of its ability to induce differentiation. In this context, we might envision that GO could be used to clear residual CSCs, with the aim of preventing tumor recurrence and distant metastasis, thereby providing a practical means for achieving “differentiation-based nano-therapy” (Fiorillo et al., 2015); **(B)** Schematic illustration of the preparation process of SAL-SWNTs-CHI-HA [chitosan(CHI) coated single wall carbon nanotubes (SWNTs) loaded with salinomycin (SAL) functionalized with hyaluronic acid (HA); Yao et al., 2014]; **(C)** Schematic diagram illustrating the concept of functionalized SWCNTs as drug carriers (Al Faraj et al., 2016b); **(D)** Left: schematic model showing surface and chemical structure of (ND) and Epirubicin (Epi), synthesis and aggregation of Epirubicin-nanodiamond complex (EPND). ND represented in truncated octahedron structure with different surface charge denoted with color. ND surface functional group indicated, including benzene ring, carboxyl group, and hydrogen group. Molecular skeleton representing carbon, oxygen and nitrogen atoms in Epi molecule was shown in red. Synthesis of EPND was performed under basic condition of 2.5 mM NaOH through physical adsorption between Epi and ND. Aggregation around 90 nm was formed after EPND synthesis. Right: representative image shows that EPND can inhibit tumor-initiation in murine hepatic tumor allografts (Wang et al., 2014).

Previous studies have shown that GO can be used for targeted cancer therapies, prevent tumor growth and inhibit tumor cell migration (Tian et al., 2011; Gonçalves et al., 2013; Gurunathan et al., 2015). In 2014, Jung et al. reported a photothermal therapy based on transdermal nano-graphene oxide-Hyaluronic acid (NGO-HA) conjugates for melanoma skin cancer by using near-infrared (NIR) laser. Because the melanoma tissues of mice are relatively leaky and express high levels of HA receptors, thus NGO-HA could easily penetrate and retain in the tumor tissues for ablating tumor efficiently without recurrence (Jung et al., 2014). However, studies that exploited GO in CSC therapy for cancer treatment is rare. Fiorillo et al. demonstrated that GO is efficient in inhibiting tumor-sphere formation in six independent cancer cell lines, across multiple tumor types (prostate, ovarian, breast, lung, pancreatic, and brain cancer). They employed the tumor sphere assay, which functionally measures the tumor sphere formation and expansion from single CSCs under anchorage-independent conditions, to evaluate the GO-targeted therapy. The obtained results suggested that GO specifically targets a global phenotypic property of CSCs and it may reduce the number of bonafide CSCs by inducing their differentiation and inhibiting their proliferation (Figure 2A, Fiorillo et al., 2015). In a word, the author here present evidence that GO based therapy may be effective in exterminating CSCs by inhibiting several key signal pathways and then inducing CSC differentiation.

## Carbon nanotubes

Carbon nanotubes are cylindrical graphene nanostructures with unique properties, such as water-solubility, cell membrane penetrability, high drug-loading ability, selective retention in the tumor, low toxicity, photothermal, photoacoustic, and Raman properties which are valuable for nanotechnology and clinical research (Shao et al., 2013; Wu et al., 2014). In 2012, Burke et al. demonstrated that breast cancer stem cells (BCSCs) are sensitive to carbon nanotube-mediated thermal treatment and lose their long-term proliferative capacity after nanotube-mediated thermal therapy (Burke et al., 2012). Therefore, the nanotube-mediated thermal treatment can simultaneously eliminate both the differentiated cells that constitute the bulk of a tumor and the BCSCs that drive tumor growth and recurrence. In 2014, a gastric CSCs-specifically targeting drug delivery system (SAL-SWNT-CHI-HA complexes) based on chitosan(CHI) coated single wall carbon nanotubes (SWNTs) loaded with salinomycin (SAL) functionalized with hyaluronic acid (HA) were fabricated by Yao et al. The constructed system was shown to have ability to selectively eliminate gastric CSCs (Figure 2B, Yao et al., 2014). Al Faraj et al. developed a strategy that employed biocompatible multimodal SWCNTs functionalized with CD44 antibodies and confirmed the enhanced selective targeting of anti-CD44, which provided encouraging results for efficient targeting of breast CSCs and perspectives for further clinical studies (Al Faraj et al., 2016a). Soon afterwards, the same group combined Paclitaxel and Salinomycin drugs conjugated SWCNTs (Figure 2C) to actively target both breast cancer and CSCs in xenograft murine model and the results confirmed the enhanced therapeutic effect of the combined therapy compared to treatment with individual drug-conjugated nanocarriers or free drug suspensions. Thus, the developed conjugated SWCNTs drug delivery system holds great promise for effective breast cancer therapy by targeting both cancer cells and CSCs (Al Faraj et al., 2016b).

## Nanodiamond

Nanodiamonds are truncated semi-octahedral carbon structures, and the surface of which can be functionalized with a wide variety of biological and chemical agents, including small molecules, therapeutic and targeting biomolecules, genetic material as well as imaging agents (Liu et al., 2009). Among a wide variety of nanomaterials-based vehicles, nanodiamonds (NDs) have shown outstanding delivery ability and excellent biocompatibility (Zhang et al., 2016). Zhao et al. have demonstrated that detonation nanodiamond with hyperbranched polyglycerol coating (dND-PG) loaded with anticancer drug and led by efficient targeting moiety can realize highly preferential toxicity to the intended tumor cells through specific uptake mechanisms, while with minimum uptake and toxicity in macrophages (Zhao et al., 2014). Nanodiamond-drug complex by physical adsorption of Epirubicin on nanodiamonds was also fabricated and was demonstrated to be a highly effective nanomedicine-based approach to overcome chemoresistance in hepatic CSCs. As shown in Figure 2D, the resulting Epirubicin@nanodiamonds complex, EPND, possesses enhanced treatment compared with unmodified Epirubicin (Wang et al., 2014). The ability to attach various bioactive molecules, including cell-specific ligands, to carbon molecules enables carbon-based nanomaterials to be an efficient solution for cancer therapy by targeting CSCs.

## DNA origami for targeting CSCs

DNA self-assembling nanostructure (Lanier and Bermudez, 2015; Kim et al., 2016; Xia et al., 2016), especially DNA origami, has been considered as the most promising candidates as a drug delivery carrier for cancer therapy (Zhao et al., 2012; Ouyang et al., 2013; Zhu et al., 2013; Zhang et al., 2014; Jiang et al., 2015; Zhuang et al., 2016). DNA origami were prepared through the self-assembly of a long single stranded M13mp18 phage DNA and hundreds of complementary short DNA staples, which endows the structure with high levels of structural programmability, obvious biocompatibility, and easiness to be modified with functional moieties. Furthermore, DNA origami can be functionalized with the agents of interest with high spatial precision, the so called “addressability.” With the aid of this technology, nanoscale assemblies of drugs and other active agents can be organized with unprecedented precision and with high load for targeted therapies.

**Figure 3:**
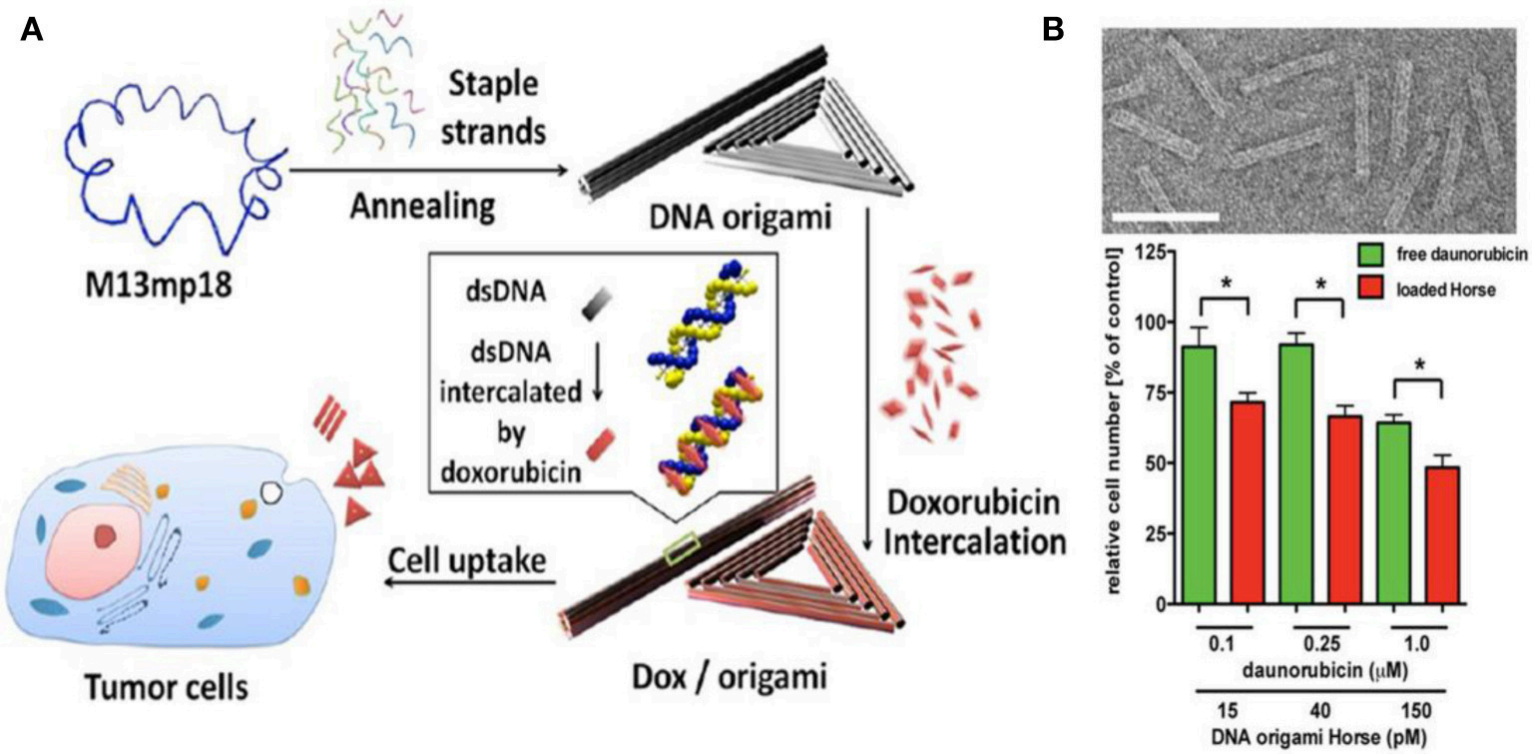
**(A)** DNA origami and doxorubicin origami delivery system assembly. The long single-strand M13mp18 genomic DNA scaffold strand (blue) is folded into the triangle and tube structures through the hybridization of rationally designed staple strands. Watson−Crick base pairs in the double helices serve as docking sites for doxorubicin intercalation. After incubation with doxorubicin, the drug-loaded DNA nanostructure delivery vessels were administered to human breast cancer cell line MCF 7 cells, and the effects were investigated (Jiang et al., 2012); **(B)** Top: Transmission electron microscope (TEM) images of the designed DNA origami structure; Bottom: The number of viable cells subjecting to free daunorubicin or daunorubicin-loaded Horse nanostructures for 24 h (Halley et al., 2016). ^*^p < 0.05.

In 2012, Jiang et al. reported a drug delivery system (Figure 3A) based on triangular and tubular DNA origami nanostructures, which are spatially addressable, of high loading capacity and good biocompatibility. Then, doxorubicin were loaded in these structures and administered to human breast cancer cell line MCF-7 cells and the effective internalization of the structure by both cell lines were confirmed by confocal fluorescent analyses. The origami-doxorubicin complex exhibited prominent cytotoxicity not only to regular human breast cancer cells (MCF 7), but more importantly to doxorubicin-resistant cancer cells, inducing a remarkable reversal of phenotype resistance. Then, the authors studied the means by which the DNA origami-drug complex circumvents resistance in res-MCF 7 cells. The results indicated that the DNA nanostructure delivery platform circumvented drug resistance in res-MCF 7 cells by increasing the cellular uptake of doxorubicin and inducing a change in lysosomal pH that redistributed the drug to target sites (Jiang et al., 2012). Halley et al. synthesized a rod-like DNA origami drug carrier (Figure 3B) that can be controllably loaded with daunorubicin and demonstrated the ability of the DNA origami-drug complex to circumvent efflux-pump-mediated drug-resistance of leukemia cells. Their results directly showed that DNA origami-based daunorubicin delivery had the potential to treat acute leukemia cells exhibiting multi-drug resistance (MDR). In addition, the results revealed that circumvention of MDR could be achieved at concentration ranges of 0.1–1.0 μmol/L daunorubicin. What's more, they found that it is crucial to control the quantities of drug loaded in origami to maximize the drug efficacy, especially in disrupting cellular proliferation (Halley et al., 2016).

Nowadays, it is simple to synthesize various DNA origamis with a series of geometric and aesthetic shapes (triangle, rectangle, pillar, and so on) with diverse dimensions (1D, 2D, and 3D) using rational design principles. In addition, the fully addressable DNA origami nanostructures can be produced in extremely high yields and a wide range of molecules and nanoparticles can be functionalized on the nanostructures through hundreds of addressable staples. What's more, it is suggested that DNA origami were of excellent stability in cell lysate, which is of great significance for drug delivery and controlled drug release. All these properties enable DNA origami structures a promising tool in biomedical fields, especially in cancer nanotechnology. We believe that DNA origami has unlimited potential and will play important roles in cancer therapies in the future.

## Gold nanoparticles for targeting CSCs

Gold nanoparticles [e.g., Au NPs and gold nanorods (Au NRs)] have been widely used in cancer research (Wang et al., 2011; Chen et al., 2012; Iodice et al., 2016) by the reason of their facile synthesis, easiness for functionalization, localized surface plasmon resonance, and excellent biocompatibility (Huang and El-Sayed, 2010). In fact, it has been well-established that Au NPs are biocompatible (non-cytotoxic and non-immunogenic), which is very important for the widespread applications in nanomedicine and drug delivery (Ghosh et al., 2008; Brown et al., 2010; Kong et al., 2016). All these properties enable them very suitable for clinical research (Lee et al., 2011; Tiloke et al., 2016). Nowadays, sphere Au NPs (Sun and Xia, 2002) of different diameters can be synthesized in high quality and high yield by the well-known citrate reduction method. The progress of synthetic chemistry in the last decade enables Au NPs of different shapes (Murphy et al., 2005; Xia et al., 2009) and sizes to be synthesized, including Au NRs (Jana et al., 2001; Nikoobakht and El-Sayed, 2003), gold nanoflowers (Xie et al., 2008; Wang et al., 2010), gold nanocages (Skrabalak et al., 2008; Xia et al., 2011), and so on. The radiative properties of gold nanoparticles including absorption, scattering and localized surface plasmon resonance (LSPR) make them very suitable for photothermal therapy and molecular cancer imaging. While the easiness for surface modification enables them very suitable for drug delivery (Tian et al., 2016) and cancer therapies.

## Sphere Au NPs

The unique physico-chemical properties of Au NPs have been used for targeted drug delivery (Ghosh et al., 2008; Elbialy et al., 2015) in almost all cancer types and have demonstrated enhanced anti-tumor efficacy (Patra et al., 2010; Wagstaff et al., 2012; Setua et al., 2014). However, the discovery of CSCs has changed the direction of the targeted chemotherapy and directs the anti-cancer research toward targeting CSCs (Atkinson et al., 2010; Sun et al., 2014a,b; Gilam et al., 2016; Yi et al., 2016).

**Figure 4:**
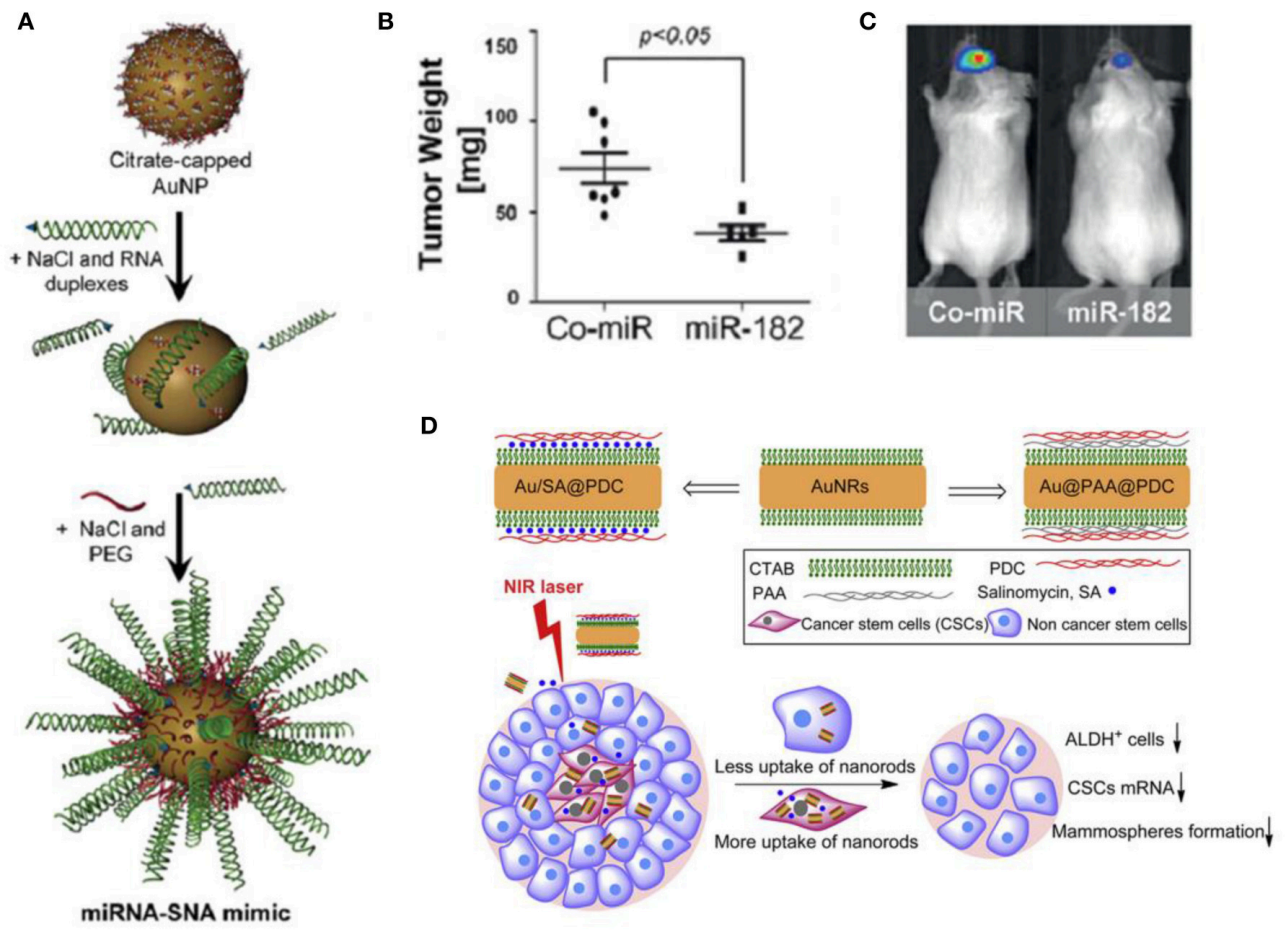
**(A)** miR-182 or Co-miR–RNA duplexes were hybridized to citrate stabilized gold nanoparticles (Au NPs) via thiol-gold bond and passivated with polyethylene glycol-Thiol (mPEG-SH) to obtain miR-182-based spherical nucleic acids (182-SNAs); **(B)** Analysis of tumor burden by weight and **(C)** bioluminescence imaging; **(A–C)** (Kouri et al., 2015); **(D)** Top: fabrication of polyelectrolyte conjugated Au NRs and drug loading; Bottom: schematic illustration of selective elimination of breast cancer stem cells (CSCs) by polyelectrolyte conjugated gold nanorods (Au NRs) mediated hyperthermia. CTAB, cetyltriethylammnonium bromide; PAA, Poly(acrylic acid); PDC, poly-diallyldimethylammonium chloride; ALDH^+^, aldehyde dehydrogenase positive (Xu et al., 2014).

Tomuleasa et al. reported a novel strategy based on functionalized Au NPs for Hepatocellular carcinoma and lowered the chemoresistance of Hepatocellular Carcinoma Cells. They first stabilized Au NPs with a monolayer of L-aspartate and then additional drugs (e.g., doxorubicin, cisplatin, and capecitabine) were conjugated through non-covalent interaction to obtain the drug complex. Tumor-targeting results suggested that the cellular proliferation in the presence of the anti-cancer drugs complex prepared from the Au NPs were repressed compared with those of cells exposed to the cytostatic drugs alone, indicating that Au NPs increased the susceptibility of Hepatocellular Carcinoma Cells to these drugs (Tomuleasa et al., 2012). Cancer cells undergo faster metabolism and consume more glucose than normal cells, taking advantage of this property, Hu et al. chose glucose as a reagent to target cancer cells. Au NPs modified with thio-PEG (polyethylene glycol) and thio-glucose (Glu-Au NPs) was created for targeted treatment of cancer metastasis and CSCs. Using human monocytic cell line derived from acute monocytic leukemia patients as a model (due to its properties are similar to CSCs), and then fed the cells with Glu-Au NPs followed by X-ray irradiation. The experimental results show that Glu-Au NPs enhanced the elimination of human monocytic cells 20% more than X-ray irradiation alone and Au NP treatment alone (Hu et al., 2015). Kouri et al. synthesized Au NPs modified with mature miR-182 duplexes [miR-182-based spherical nucleic acids (182-SNAs), Figure 4A] and injected 182-SNAs intravenously to the orthotopic Glioblastoma multiforme (GBM) xenografts. The results showed that 182-SNAs could penetrate the blood–brain/blood–tumor barriers and selectively disseminate throughout extravascular glioma parenchyma, leading to shrinked tumor size and increased survival rates (Figures 4B,C). The authors, here, present a novel strategy for therapeutic intervention in GBM by exploiting the anti-tumor activities of miR-182 which was modified on Au NPs to form spherical nucleic acids (Kouri et al., 2015).

## Gold nanorods (Au NRs)

Compared with Au NPs, Au NRs have greater advantages in cancer cell imaging and photothermal therapy based on their localized surface plasmon resonance (LSPR) at near-infrared (NIR) wavelength band (Oli, 2010; Peng and Wang, 2011). One of the advantages of Au NRs is that the light source used for imaging and photothermal therapy can be NIR wavelength band which induce less damage and possess better tissue penetrability. Therefore, Au NRs has been widely used in cancer research, especially in photothermal therapy (Amreddy et al., 2015; Liu et al., 2016).

Wang et al. reported a strategy by using aptamer-modified Au NRs for targeted photothermal therapy of prostate CSCs. In this work, two kinds of aptamers [against DU145 prostate cancer cells (aptamer CSC1) and against prostate CSCs (aptamer CSC13)] were modified on the surfaces of Au NRs, and the obtained Au NRs complex successfully targeted and destructed both cancer cells and CSCs through NIR laser irradiation (Wang et al., 2013). Xu et al. found that photothermal therapy mediated by Au NRs can selectively eliminate breast CSCs (Xu et al., 2014). The results suggested that polyelectrolyte conjugated Au NRs treatment reduced the aldehyde dehydrogenase positive (ALDHþ) cells subpopulation, the gene expression of stem cell markers and the mammosphere forming ability. Cellular uptake assay suggested that one of the possible reasons for the selective elimination of CSCs is they could internalize much more and faster of the conjugated Au NRs. The authors further combined the chemotherapy and photothermal therapy, loading the polyelectrolyte conjugated Au NRs with salinomycin (SA), and obtained enhanced inhibition of CSCs (Figure 4D).

**Figure 5:**
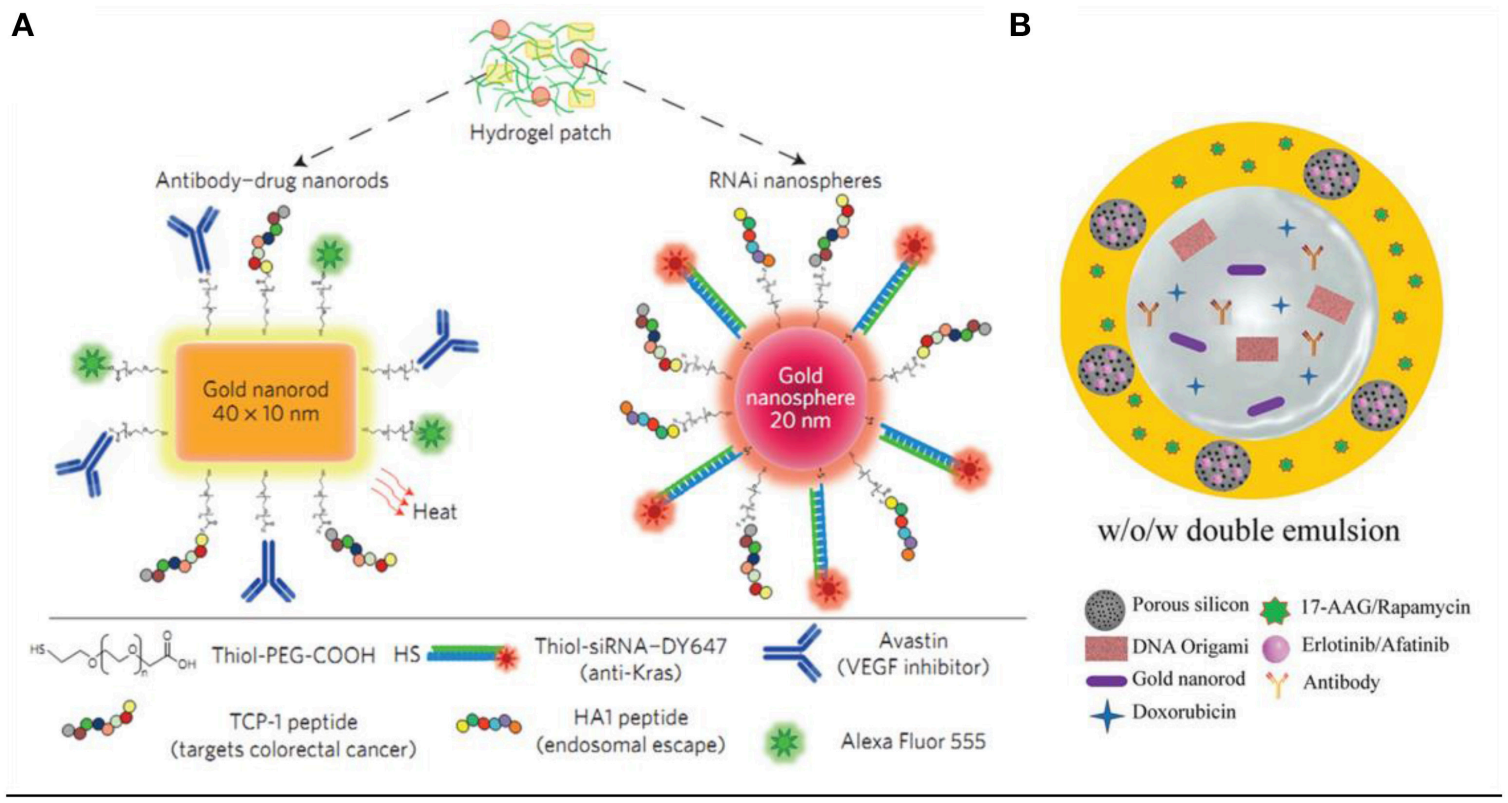
**(A)** Drug–gold nanorods and siRNA–gold nanospheres doped in implantable hydrogels for local drug/gene delivery and local hyperthermia (Conde et al., 2016). **(B)** Schematic illustration of the preparation of biocompatible porous silicon nanoparticles@gold nanorods@double emulsion (PSi NPs@AuNRs@double emulsion) co-delivery platform for co-loading versatile therapeutics, DNA origami, antibody, and hydrophobic functional PSi NPs loaded with Erlotinib or Afatinib (Kong et al., 2016).

The combination of multiple therapies for cancer treatment has already been inevitable and is also a general tendency. The development and integration of materials science, bioimaging, and cancer biology has now enabled the design of stimuli-responsive intelligent platforms for cancer therapies (Conde et al., 2015). In 2016, Conde et al. developed a triple-integration therapy, a combination of gene therapy, drug therapy and photothermal therapy, to remit non-resected tumor, and prevent tumor recurrence after the tumor excision surgery (Conde et al., 2016). They loaded hydrogel with Au NRs@drug (for chemotherapy and photothermal therapy) and Au NPs@siRNA (for Kras gene silencing) for triple therapies (Figure 5A), while the hydrogel was used to stabilize the integral delivery system and enable local delivery of the conjugates. In the same year, Kong et al. prepared a biocompatible double emulsion system with higher integration degree, which integrated porous silicon nanoparticles (PSi NP), Au NRs, DNA origami, antibody, doxorubicin, 17-AAG or Rapamycin, and Erlotinib or Afatinib in one platform; (Kong et al., 2016; Figure 5B). The all-in-one system could overcome multidrug resistance and enable more effective treatments of cancer, thus it holds great potential in biomedical field for cancer therapy.

Au NPs are potential nanomaterials to be utilized in CSCs targeting for the following reasons: firstly, they can be easily synthesized a wide variety of sizes and shapes; secondly, they are very stable, of highly biocompatibility and easily to be functionalized with various bioactive agents; Thirdly, they possess unique optical properties and are easily tracked and detected by absorption/scattering spectroscopy.

## Organic nanoparticles for targeting CSCs

Liposomes, polymeric nanoparticles, and dendrimers have been the most widely studied carriers in the field of nanoscale drug delivery (Duncan and Gaspar, 2011). Why they are so popular? First, they hold high stability and good biocompatibility both *in vitro* and *in vivo*, and can solubilize a wide range of poorly soluble drugs. Polymeric nanoparticles are commonly prepared from natural polymers (such as chitosan) or synthetic biocompatible polymers [such as poly-lactic-co-glycolic acid (PLGA)], while liposomes, analogs of biological membranes, have always been regarded as one of the most biocompatible vehicles for drug delivery (Colson and Grinstaff, 2012; Hadinoto et al., 2013; Mandal et al., 2013; Crucho, 2015). In addition, polyethylene glycol (PEG) is usually conjugated to the polymer nanoparticles to enhance the immune-compatibility. Second, they can avoid the short time drug degradation after administration. Third, they can also prevent undesirable side effects on normal cells, organs, and tissues by some cytotoxic drugs. The last but not the least, they can increase drug bioavailability and the fraction of the drug accumulated in the pathological area. A variety of drug delivery and drug targeting systems, such as synthetic polymers(Chenna et al., 2012; Usacheva et al., 2014; Kumar et al., 2015), microcapsules (Chen et al., 2015), lipoproteins (Helbok et al., 2012; Shen et al., 2016), liposomes (Yuan et al., 2013; Han et al., 2014; Lokerse et al., 2016), lipid particles(You et al., 2015), and many others have been designed and exploited for cancer therapy (Torchilin, 2006). Therefore, they hold great potential to generate practical strategies for the CSC therapy in the near future.

## Liposome

A liposome is a spherical vesicle that composed of at least one lipid bilayer and it can be used as a vehicle for delivering drugs. Liposomes can ameliorate the stability and pharmacokinetics of free drugs and furthermore improve the safety and efficiency of them, but the therapeutic efficacy of them has not been sufficiently enhanced. Compared with non-targeted liposome, targeted treatment of cancer cells, especially the CSCs, do hold great potential to improve the therapeutic index, and decrease the influence of off-target phenomenon.

**Figure 6:**
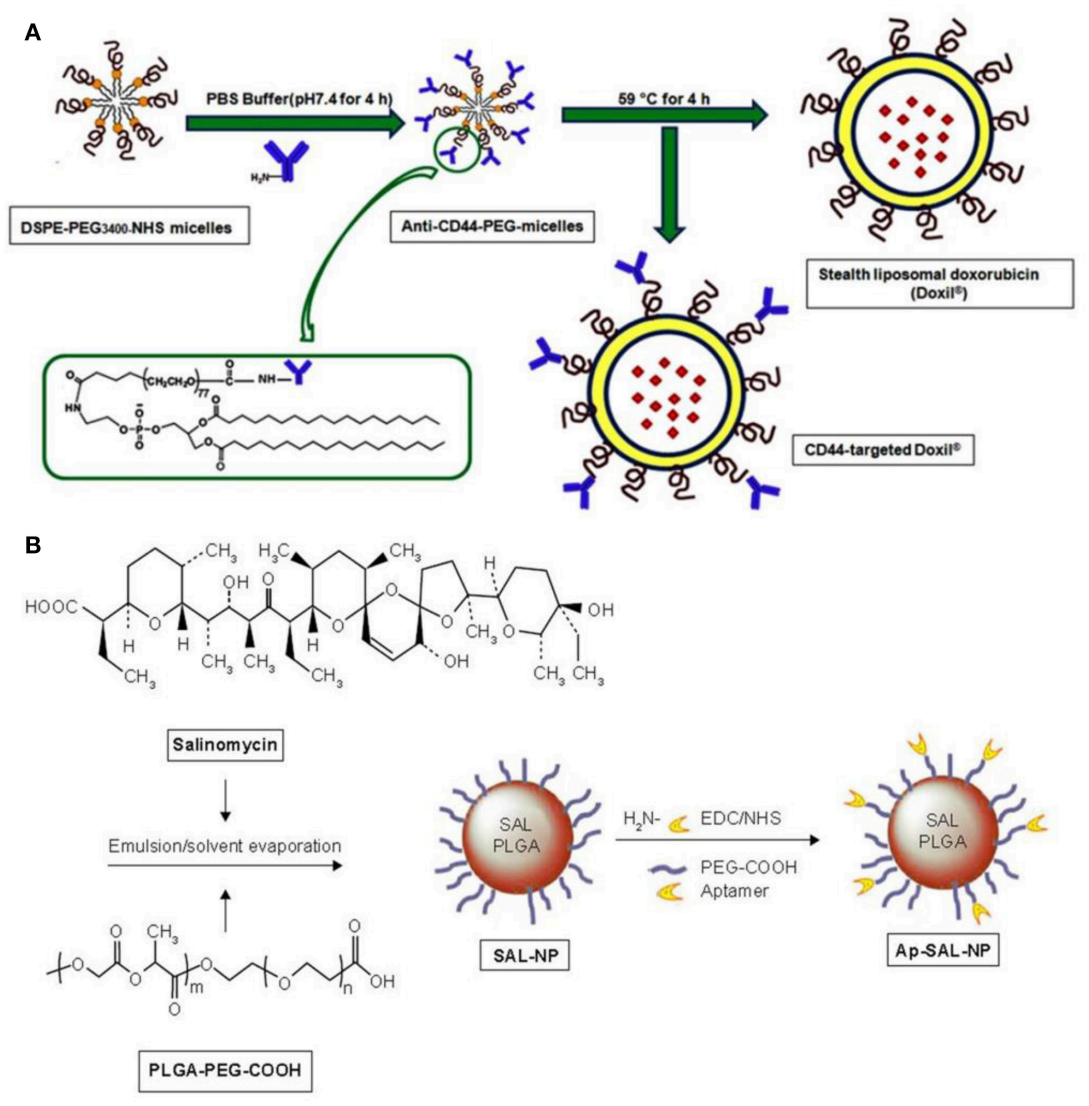
**(A)** Post-insertion method for the preparation of CD44-doxil (Arabi et al., 2015). **(B)** The preparation procedure of salinomycin-loaded PEGylated poly(lactic-co-glycolic acid) nanoparticles (SAL-NP) or SAL-NP linked with CD133 aptamers (Ap-SAL-NP; Ni et al., 2015).

Liu et al. first synthesized a liposome involving antialcoholism drug disulfiram (shorted for lipo-DS) combined with copper *in vivo*, aiming to target CSCs and avoid pan-chemoresistance (Liu et al., 2014). Lipo-DS targeted NFκB pathway, that promote hypoxia-induced CSCs and these fabricated Lipo-DS/CuGlu (copper gluconate) showed a strong anti-CSC efficacy. In the following year, Shen et al. fabricated a novel Nano-Taxol (encapsulated paclitaxel in liposome), and then investigated its effects on the stem ness phenotype and metabolic reprogramming of CSC (Shen et al., 2015). They found that intraperitoneal administration of Nano-Taxol influenced the metabolic reprogramming of cells, from glycolysis to oxidative phosphorylation and effectively suppressed CSCs. Compared with intravenous delivery of Taxol? (current standard treatment), Nano-Taxol showed a significantly better control of tumor growth. This research may provide a new approach for the nanomedicine development. In the near future, this method can be applied to the treatment of several relevant cancers that have been proved to be suitable for local delivery of therapeutic agents, including colon cancer, gastric cancer, and pancreatic cancer. In 2015, Basak et al. demonstrated that delivery of Curcumin-difluorinated (CDF) liposomes was a useful method for cisplatin resistant Head and neck squamous cell carcinoma (HNSCC) therapy (Basak et al., 2015). CDF, synthesized from the curcumin and wrapped with liposomes, was applied to evaluate the growth inhibition of cisplatin resistant HNSCC cell lines CCL-23R and UM-SCC-1R, and showed significant growth inhibition in these drug-resistant cell lines. Then, Arabi et al. constructed monoclonal antibody (mAb) modified doxil (Figure 6A), which would not damage the biodistribution of a long-circulating carrier, and used it to target CD44, one of the most well-known surface markers related with CSCs. The result indicated the potential of anti-CD44 mAb in the improvement CSC therapy (Arabi et al., 2015).

ANV-1 was a liposomal formulation for carrying anticancer drug to breast cancer stem-cell-like cells, and its pharmacokinetics in an animal model also had been evaluated. The anticancer drug ESC8 connected with dexamethasone (Dex)-associated liposome (DX) to form ESC8-entrapped liposome named DXE. The results showed DXE was a promising liposomal formulation with potent pharmacokinetic and tumor regressing profile that could sensitize and kill highly aggressive and drug-resistive tumor progenitor cells (Ahmad et al., 2016). Since liposomes provide a biocompatible and biodegradable container for loading drugs and the surface of which can be modified with various targeting ligand, they hold great possibility to deliver drugs for targeted cancer therapy.

## Polymer nanoparticles

Targeting cancer metabolism is emerging as a successful strategy for cancer therapy. In 2012, Xu et al. constructed NanoHHI (nanoparticle-encapsulated inhibitor of the hedgehog transcription factor) by the oil-in-water (o/w) emulsion solvent evaporation method that loaded PLGA-PEG (poly(lactic-co-glycolic acid) - polyethylene glycol) nanoparticles with HPI-1 (Xu et al., 2012). NanoHHI significantly suppressed the growth of both Huh7 and MHCC97L cells, decreased the population of CD133-positive hepatocellular carcinoma cells (HCC). Thioridazine (THZ), which was reported to have ability to kill CSCs, was combined with doxorubicin (DOX) to eradicate both cancer cells and DOX-resistant CSCs to mitigate the cancer relapse (Ke et al., 2014). The micelles were self-assembled from a mixture of acid-functionalized poly (carbonate) and poly(ethylene glycol) diblock copolymer (PEG-PAC) and urea-functionalized poly(carbonate) (PUC) and PEG diblock copolymer (PEG-PUC). Co-delivery of free DOX and THZ with the micelles showed strong inhibitory effect against cancer cells and CSCs. This combination therapy can target both cancer cells and CSCs offered a favorable strategy for breast cancer therapy. In 2015, Ni et al. developed nomycin-loaded nanoparticles to eliminate CD133+ osteosarcoma CSCs (Ni et al., 2015). Then, salinomycin-loaded PEGylated poly (lactic-co-glycolic acid) nanoparticles (SAL-NP) connected with CD133 aptamers (Ap-SAL-NP) (Figure 6B) were constructed by the method of emulsion/solvent evaporation. The results suggested that CD133, a well-known surface marker in CSCs, was a prospective target for drug delivery to osteosarcoma CSCs and that it is potential to significantly inhibit the osteosarcoma growth by killing CD133+ osteosarcomal CSCs.

In 2013, Swaminathan et al. synthesized poly(D,L lactide-co-glycolide) nanoparticles modified with anti-CD133 monoclonal antibody and paclitaxel, a microtubule-stabilizing anticancer agent, to target CD133+ cells (Swaminathan et al., 2013). CD133-targeted nanoparticles (CD133NPs) were effectively engineered to target cells which abundantly express CD133, and the NPs show significant suppression of Caco-2 cells, decreasing the number of mammospheres and colonies formed compared with the free paclitaxel treatment. Nanogel-drug conjugates based on membranotropic cholesteryl-HA (CHA) were also developed by Wei et al. for efficient targeting and suppressing drug-resistant tumors (Wei et al., 2013). The conjugates significantly increased the solubility and bioactivities of poorly soluble drugs, such as etoposide, salinomycin, and curcumin against CSC. These nanogels were efficiently internalized via CD44 receptor and was shown to be capable of penetrate multicellular cancer spheroids and displayed higher cytotoxic effect in the system modeling tumor environment than both free drugs and HA-drug conjugates. Cationic lipid-assisted poly(ethylene glycol)-b-poly(d,L-lactide) (PEG-PLA) nanoparticles, can efficiently deliver siRNA into U87MG and U251 glioma stem cells and bulk glioma cells, simultaneously inhibited the self-renewal of these cells in a glucose restricted tumor micro-environment. PEG-PLA Nanoparticles with specific siRNA targeting GLUT3 (NPsiGLUT3) significantly reduced the GLUT3 expression in glioma stem cells and bulk glioma cells, and also inhibited the metabolism, proliferation, and downregulated further glioma stem cells percentage (Xu C. F. et al., 2015). Because of the diversity of organic nanoparticles and the tunability of their properties, they have been exploited extensively for cancer therapies and will play a bigger role in the future.

## Summary and outlook

CSCs, also called as cancer- (or tumor-) initiating cells, are not only a grand challenge in cancer therapy, but also a great opportunity for researchers to overcome cancer. CSCs have been regarded as one of the most possible reasons for the inefficiency or failure (recurrence and metastasis) of current cancer therapies, which can be mainly ascribed to the multidrug resistance, dormancy and resistance to apoptosis properties of these cells. All these properties enable them obvious and challenging targets for improving the present therapeutic approaches. Despite the challenges, much effort has been devoted to selectively target CSCs. A wide spectrum of materials, such as carbon, DNA, metal, polymer has been used in cancer therapy by targeting CSCs as summarized in Table 1. Each of these materials has its own unique properties, such as high levels of structural programmability and addressability of DNA origami, stability, and tunable optical properties of Au NPs, Electro-conductivity and large surface area of grapheme and diversity of polymer NPs, combine several of these materials and take advantage of these properties will provide better solutions for the targeted and controlled destruction of CSCs.

**Table 1:** **The list of nanoparticles targeting CSC**.

**Type of nanoparticles**		**Targets**	**Anticancer agent**	**Type of cancer**	**References**
Carbon nanomaterials	Graphene oxide	Six independent cancer cell lines, HA receptors	Nanographene oxide, nanographene oxide-Hyaluronic acid	Breast cancer, ovarian cancer, prostate cancer, lung cancer, pancreatic cancer, brain cancer, melanoma skin cancer	Jung et al., 2014; Fiorillo et al., 2015
	Carbon nanotubes	HA receptors, CD44	Carbon nanotube, Paclitaxel/SAL(salinomycin)-SWNT(single wall carbon nanotubes)-CHI(chitosan)-HA(hyaluronic acid)	Breast cancer stem cells, gastric cancer stem cells	Yao et al., 2014; Al Faraj et al., 2016a,b
	Nanodiamond	None	Nanodiamond with hyperbranched polyglycerol with anticancer drug (dND-PG-Anticancer drug), Epirubicin@nanodiamonds(EPND)	Hepatic cancer stem cells	Wang et al., 2014; Zhao et al., 2014
DNA origami	Triangle, tube etc.	None	Doxorubicin-DNA nanostructure delivery platform, rod-like DNA origami daunorubicin carrier	Human breast cancer cells (MCF 7), doxorubicin-resistant cancer cells, leukemia cells exhibiting multi-drug resistance (MDR)	Jiang et al., 2012; Halley et al., 2016
Gold nanoparticles	Sphere Au NPs	None	Monolayer L-aspartate Au NPs with drugs, Au NPs modified with thio-PEG (polyethylene glycol) and thio-glucose (Glu-Au NPs), Au NPs modified with mature miR-182 duplexes	Hepatocellular carcinoma, cancer metastasis and cancer stem cells, Glioblastoma multiforme (GBM)	Tomuleasa et al., 2012; Hu et al., 2015; Kouri et al., 2015
	Gold nanorods	TGF-β, acid-liable, ALDH	Polyelectrolyte conjugated Au NRs with salinomycin (SA)	Aldehyde dehydrogenase positive (ALDHþ) cells	Wang et al., 2013; Xu et al., 2014
Organic nanoparticles	Liposome	NFκB pathway, CCL-23R, UM-SCC-1R, CD44	Lipo-DS/CuGlu, Nano-Taxol, Curcumin-difluorinated (CDF) liposomes, monoclonal antibody (mAb) modified doxil, ESC8 dexamethasone liposome (DXE)	Colon cancer, gastric cancer, pancreatic cancer, cisplatin resistant Head and neck squamous cell carcinoma (HNSCC), breast cancer stem-cell, aggressive and drug-resistive tumor progenitor cells	Liu et al., 2014; Arabi et al., 2015; Basak et al., 2015; Shen et al., 2015; Ahmad et al., 2016
	Polymer nanoparticles	CD133+, CD44	Nanoparticle-encapsulated inhibitor of the hedgehog transcription factor(NanoHHI), acid-functionalized poly(carbonate) and poly(ethylene glycol) diblock copolymer (PEG-PAC) and urea-functionalized poly(carbonate) (PUC) and PEG diblock copolymer (PEG-PUC), CD133-targeted nanoparticles (CD133NPs), Cationic lipid-assisted poly(ethylene glycol)-b-poly(d,L-lactide) (PEG-PLA) nanoparticles with specific siRNA targeting GLUT3 (NPsiGLUT3)	Huh7 and MHCC97L cells, hepatocellular carcinoma cells (HCC), doxorubicin resistant cancer stem cells, CD133+ cells, Caco-2 cells, U87MG and U251 glioma stem cells and bulk glioma cells	Xu et al., 2012; Swaminathan et al., 2013; Wei et al., 2013; Ke et al., 2014; Ni et al., 2015; Xu C. F. et al., 2015

The biological structures and functionalities of cancer cells, especially CSCs, are very complicated, thus it is essential to explore the exact mechanisms, to further understand their cell biology, and, most importantly, to find biomarkers and pathways for the specific targeting and destructing the CSCs. In a word, the ultimate goal of CSC research is to identify effective targeting biomarkers, delivering pathways, and therapeutics that can eliminate CSCs of various cancers, which may be realized by combining advances in cell biology of CSC and progress in nanotechnologies. In the future, multifunctional nanosystems would be a solution for the early detection and destruction of CSCs.

## Author contributions

WQ and GH wrote the manuscirpt, ZC and YZ revised the manuscript. WQ and GH contributed equally to this review.

## Funding

This work was supported by the National Natural Science Foundation of China (Nos. 81601571, 31370868 and 21375152), a Start-up Grant from Sun Yat-Sen University and the Fundamental Research Funds for the Central Universities (No. 16ykzd13).

### Conflict of interest statement

The authors declare that the research was conducted in the absence of any commercial or financial relationships that could be construed as a potential conflict of interest.
